# Rationale and Design of a Statewide Cohort to examine efficient resource utilization for patients with Intracerebral hemorrhage (EnRICH)

**DOI:** 10.1186/s12883-018-1036-1

**Published:** 2018-03-21

**Authors:** Farhaan S. Vahidy, Ellie G. Meyer, Arvind B. Bambhroliya, Jennifer R. Meeks, Charles E. Begley, Tzu-Ching Wu, Jon E. Tyson, Charles C. Miller, Ritvij Bowry, Wamda O. Ahmed, Gretchel A. Gealogo, Louise D. McCullough, Steven Warach, Sean I. Savitz

**Affiliations:** 1 Department of Neurology and the Institute for Stroke and Cerebrovascular Disease, McGovern Medical School, University of Texas - Health, Houston, TX USA; 2 Department of Management, Policy, and Community Health, School of Public Health, University of Texas Health Science Center at Houston, Houston, TX USA; 3 Center for Clinical Research and Evidence Based Medicine at McGovern Medical School, University of Texas Health Science Center at Houston, Houston, TX USA; 4 Department of Neurosurgery, McGovern Medical School, University of Texas Health Science Center at Houston, Houston, TX USA; 5 Department of Neurology, Dell Medical School, The University of Texas at Austin, Austin, TX USA

**Keywords:** Stroke, Intracerebral hemorrhage, Patient-centered outcomes, Quality of life, Readmission rate, Mortality, Cost effectiveness, Resource utilization

## Abstract

**Background:**

Intracerebral hemorrhage is a devastating disease with no specific treatment modalities. A significant proportion of patients with intracerebral hemorrhage are transferred to large stroke treatment centers, such as Comprehensive Stroke Centers, because of perceived need for higher level of care. However, evidence of improvement in patient-centered outcomes for these patients treated at larger stroke treatment centers as compared to community hospitals is lacking.

**Methods / design:**

“*Efficient Resource Utilization for Patients with Intracerebral Hemorrhage (EnRICH)*” is a prospective, multisite, state-wide, cohort study designed to assess the impact of level of care on long-term patient-centered outcomes for patients with primary / non-traumatic intracerebral hemorrhage. The study is funded by the Texas state legislature via the Lone Star Stroke Research Consortium. It is being implemented via major hub hospitals in large metropolitan cities across the state of Texas. Each hub has an extensive network of “spoke” hospitals, which are connected to the hub via traditional clinical and administrative arrangements, or by telemedicine technologies. This infrastructure provides a unique opportunity to track outcomes for intracerebral hemorrhage patients managed across a health system at various levels of care. Eligible patients are enrolled during hospitalization and are followed for functional, quality of life, cognitive, resource utilization, and dependency outcomes at 30 and 90 days post discharge. As a secondary aim, an economic analysis of the incremental cost-effectiveness of treating intracerebral hemorrhage patients at higher levels of care will be conducted.

**Discussion:**

Findings from EnRICH will provide much needed evidence of the effectiveness and efficiency of regionalized care for intracerebral hemorrhage patients. Such evidence is required to inform policy and streamline clinical decision-making.

## Background

### Introduction

Intracerebral Hemorrhage (ICH) is a devastating disease. Although ICH accounts for about 10–15% of all stroke subtypes in the United States (US), this proportion is reported to be as high as 50% in certain regions of the world [1]. The burden of disease imposed by ICH is tremendous. Approximately 50% of ICH patients do not survive beyond 30 days, and up to 80% fail to achieve functional independence [2, 3]. Furthermore, approximately 90% of ICH patients report their quality of life (QOL) to be below average, including 20% stating their QOL to be worse than death [4]. There are no class I evidence-based modalities that improve ICH outcomes. The mainstay of management revolves around hemodynamic stabilization, neurological monitoring, blood pressure titration, reversal of coagulopathy (if indicated), surgical interventions (in selected patients), secondary stroke prevention, and rehabilitation [5]. It has also been reported that the majority of hospitals do not have well-developed ICH management protocols [6]. Clinical trials for early blood pressure management and surgical interventions in ICH patients have demonstrated safety with limited and equivocal efficacy [7, 8].

There are national and regional data showing that a large number of ICH patients are transferred from smaller community hospitals to bigger stroke treatment centers, probably for a perceived need of higher level of care (HLOC) [9, 10]. Data also suggest that the proportion of transferred patients to large hospitals has been increasing periodically [10]. However, the impact of management of ICH patients at a certain level of care on long term functional, cognitive, and quality of life outcomes within the current paradigm of stroke care delivery in the US is not understood. Furthermore, a subset of ICH patients that would optimally benefit from transfer to or management at centers with HLOC, such as Comprehensive Stroke Centers (CSCs), has not been identified. Finally, the comparative effectiveness of treating ICH patients at higher-resourced centers has not been provided. The study “*Efficient Resource Utilization for Patients with Intracerebral Hemorrhage (EnRICH)*” has been designed and is being implemented to provide evidence to address these important issues.

### Rationale

The American Heart Association / American Stroke Association guidelines for management of ICH were updated in 2015. These guidelines added a new Class I, level B evidence stating, “*Initial monitoring and management of ICH patients should take place in an intensive care unit or dedicated stroke unit with physician and nursing neuroscience acute care expertise*” [11]. The guidelines provide reference to three non-randomized studies as the basis for this evidence.

The first reference is made to a retrospective comparative analysis of 1038 ICH patients admitted to a general (non-neurological) intensive care unit (ICU) and those admitted to a neurological ICU (NICU) [12]. The data were collected under Project Impact that began in 1996. The duration of data collection for patients included in this study is not clear; however, since the study was published in 2001 it is assumed that the 3 years of included data are between 1996 and 2001. The study reported an odds ratio (95% Confidence Interval [CI]) of 3.43 (1.65–7.60) for inpatient mortality among ICH patients admitted to a general ICU as compared to NICUs. The authors did acknowledge that limited data from NICUs (only two were included), and voluntary participation of hospitals in the project can lead to selection bias and lack of generalizability. However, it is also pertinent to note that analyses were done at the patient level, and clustering of patients within ICUs and NICUs was not accounted for.

The second reference is made to a cross-sectional analysis of data from 49 acute care hospitals in Alberta, Canada, including 18,142 patients with diagnoses of acute myocardial infarction, congestive heart failure, chronic obstructive pulmonary disease or stroke between April 1, 1998 and March 31, 1999 [13]. The analyses were conducted with the aim of evaluating the association between nurse education and skill, continuity of care, quality of the work environment, and 30-day all-cause mortality. The authors correctly identified certain limitations pertaining to the nature of administrative data. However, it is apparent that the patient population included in this study does not allow generalizability of the results to ICH patients. Diagnosis-based stratified analyses have not been presented, and the only reported metric for stroke patients (presumably including ischemic stroke patients) is a 1% difference in 30-day mortality (15% vs. 16%) between patients discharged from low vs. high volume hospitals.

The third observational study is an analysis of the data from the Swedish Stroke Register between 2001 and 2005 [14]. It compares the risk of death, institutionalization, and dependency between 105,043 stroke patients managed at stroke units and those managed at other types of units. The authors report a benefit for ICH patients managed in stroke units in terms of mortality and dependence. However, the study does not describe the differences between stroke and other units (primary exposure in this case) in care parameters for stroke patients. More importantly, the wider generalizability of such findings from a homogenous health care system remain questionable.

It is clear that the evidence cited in the guidelines falls short in multiple domains. These studies were not conducted during the last decade and they include data from non-stroke and non-ICH patients. Furthermore, their generalizability is highly questionable – particularly to the current paradigm of stroke care delivery and stroke care certification of hospitals in the US. Finally, they largely address broad and short-term outcomes such as inpatient and 30-day mortality. These outcomes, though important administratively, may not be patient-centered.

More recent and contextually relevant evidence, evaluating the association between level of care and outcomes in ICH patients, does not clearly highlight the benefit of managing all ICH patients at a HLOC. Two analyses independently conducted at large CSCs showed that transferred patients had milder disease severity and did not significantly utilize more CSC specific treatment modalities [9, 15]. However, these data are limited by lack of outcomes for patients who are not transferred to CSCs. A direct comparison between ICH Medicare beneficiaries treated at Primary Stroke Centers (PSC) and those treated at non-certified centers did reveal a reduction in 30-day mortality for PSC patients; however, there were no differences in 30-day readmission [16]. This analysis did not consider differences between CSCs and PSCs, lacked patient-centered outcomes, and excluded patients < 65 years of age, which may constitute a considerable proportion of ICH patients. A relatively recent comparison of brain hemorrhage patients treated at CSCs vs. PSCs did not reveal any differences in 90-day mortality for ICH patients [17].

Furthermore, there are limited and conflicting data on the comparative effectiveness of ICH patient transfer for HLOC. A recent simulation-based study provided comparative effectiveness in terms of incremental cost effectiveness ratios of $47,431 per Quality Adjusted Life Years (QALY) for transferring ICH patients to a NICU under a most favorable scenario. However, these estimates are about 93% to 700% higher for less favorable scenarios [18]. Reliance on probabilistic assumptions behind simulation models, and interpretation of willingness-to-pay thresholds remain significant limitations of such analyses. Other studies based on primary data have reported that cost effectiveness of transfer of ICH patients is not clearly demonstrable, and that an evidence-based triage algorithm for optimal selection of patients is warranted [19].

Based on the above discussion, there is enough equipoise for EnRICH, which has the following aims:

1. To examine the association between level of care (as defined by certification status of the hospital) and outcomes in ICH patients
2. To estimate the health economic impact of management of ICH patients at CSCs, as compared to PSCs and / or non-certified hospitals
3. To characterize a subset of ICH patients that optimally benefits from care provision and management at HLOC (such as CSCs)

## Methods / design

### Study design and setting

EnRICH is designed to be an observational, prospective, multisite cohort study. It will be simultaneously operationalized at five major hubs in the state of Texas under the auspices of the Lone Star Stroke (LSS) Research Consortium of Texas. The LSS Research Consortium is a collaborative stroke research effort supported by the Texas legislature. Its mission is to conduct patient-centered stroke research across the state of Texas, with a specific focus on involving communities that generally do not have access to research, by utilizing the hub and spoke infrastructure of participating sites. One of the specific goals of the LSS charter is to “*Develop and disseminate evidence-based guidelines for safe, high-quality, and cost-effective stroke care in Texas*”. The LSS network provides an ideal infrastructure to fulfill the aims of the EnRICH study, and the EnRICH study directly supports the stipulated goal of the LSS consortium’s charter. Each LSS hub is a large, academic stroke treatment center designated as a CSC in most cases. A hub has a network of multiple other spoke hospitals, which are connected to the hub in their functionality via traditional arrangements or telemedicine technology. The spokes have varying patterns of transferring ICH patients to the hub. Some spokes transfer most or all ICH patients, whereas others tend to manage ICH patients in-house. Other spokes have varying proportions of transfer / non-transfer ICH patients. The details of the network and participating sites are publically available [20]. This framework provides an opportunity to enroll ICH patients who directly present either to CSC or non-CSC hospitals, and are either transferred or not transferred for HLOC.

### Study population

All male and female adult patients (≥ 18 years), of all races and ethnicities are eligible to be enrolled in EnRICH if they have a radiologically confirmed ICH (usually requiring only a non-contrast head computed tomography (CT) scan) with an etiology such as hypertension, coagulopathy or cerebral amyloidosis. We initially excluded ICH patients presenting to an index hospital 24-h after symptom onset or last seen normal, however this exclusion criteria has been removed. In our experience, decisions made at non-CSC centers to transfer radiologically confirmed ICH patients to a higher level of care are not influenced by time lapse between onset of symptoms and presentation. Among the population of transferred patients, only those patients who transfer within 24 h of their initial presentation are enrolled. Although patients with intraventricular extension of an intracerebral hemorrhage are eligible, patients with a pure or primary intraventricular hemorrhage are excluded. These patients may benefit from ventricular drainage procedures along with neurological monitoring in CSCs or other larger centers. Similarly, patients with other forms of intracranial hemorrhages, such as subdural and subarachnoid, are excluded. Patients with secondary causes of intracerebral hemorrhage - such as neoplasm, arteriovenous malformation, cerebral aneurysm, or hemorrhagic transformation of ischemic stroke - are also excluded. Secondary causes of hemorrhage are determined based on diagnostic work-up during the course of hospitalization. Potentially eligible patients are screened daily upon admission. Patients with non-primary ICH are excluded. Patients with unclear etiology are not approached, but are monitored for their diagnostic work-up and are only approached if secondary causes of ICH are excluded. If diagnostic work-up determines a secondary etiology after enrollment, patients are removed from the study. In such cases patients / their care-givers and the institutional review board (IRB) is duly notified. Detailed screening and enrollment are being maintained that track every potential ICH patient presented at an enrolling site. An IRB approved protocol amendment allows us to also enroll eligible ICH patients who die in-hospital prior to being approached for consent.

### Study procedures

**Fig. 1:**
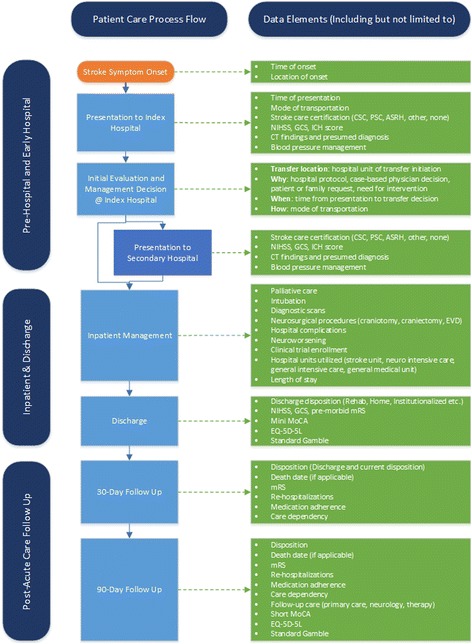
Schematic representation of patient flow through various network hospitals and data elements captured at each stage. Various study phases are indicated vertically

Eligible ICH patients with radiological confirmation of a parenchymal hemorrhage are approached to consent during their hospitalization. EnRICH is currently approved by the University of Texas Health Science Center at Houston (UTHealth) Institutional Review Board (IRB) to obtain a verbal consent from either the patient or their legally authorized representative. Upon consent, several baseline assessments are completed with the patient or by proxy, and certain preliminary information about patients – including demographic, risk factors, and initial presentation variables - is captured in the study-specific, online, secure database. Information describing inpatient management, such as treatment intensity, length of stay, procedures, laboratory investigations, and diagnostic modalities, are also captured. After discharge from acute care, consented patients are followed at 30 and 90 days via phone calls to obtain information on functional, QOL, cognitive, resource utilization, and level of dependency outcomes. A schematic representation of patient flow and information collected for EnRICH at various study phases is presented in Fig. 1.

### IRB approvals and multisite implementation

The University of Texas Health Science Center at Houston IRB is the IRB of Record. The project has been approved at various study sites via IRB reciprocity, and we aim to maintain single IRB review in compliance with the current regulatory trend. An electronic study master file has been designed and implemented to facilitate inter-site communication and centralized storage of essential study documents.

### Outcomes

The 90-day functional status as assessed by modified Rankin Scale (mRS) is the primary outcome. Secondary outcomes include 30-day readmission and 30-day mortality. The outcome for economic analysis is the relative cost effectiveness of ICH patient management at varying levels of care (i.e. CSCs vs. non-CSC hospitals). Cost effectiveness ratios are comprised of Cost (as determined by total costs of inpatient treatment intensity, transfer status, and post-discharge resource utilization weighted by Medicare reimbursement amounts) and effectiveness (as obtained by QALYs determined by EQ-5D QOL information). The exploratory outcomes include assessment of patients’ health utility as captured by Standard Gamble, cognitive outcome determined using mini Montreal Cognitive Assessment (MoCA), and change in patient dependency status.

### Statistical analysis

The primary analytical aim of EnRICH is to compare 90-day functional outcomes between ICH patients treated at CSCs and those treated and non-CSC hospitals. We propose to conduct a shift analysis of the seven point mRS (0–6) utilizing proportional odds models. Baseline variables will be assessed for imbalance between the two groups, and statistically significant (*p* < 0.1) potential confounders will be included in the multivariable models. Clinically important variables that are known to predict outcome in ICH patients (such as age, presentation National Institutes of Health Stroke Scale (NIHSS), presentation Glasgow Coma Scale (GCS), systolic blood pressure, hematoma volume, hemorrhage location, and hematoma expansion) will be included in the multivariable model regardless of their statistical significance. Model building, variable selection, and assessment of the fit of the model will be conducted utilizing methodologies described in standard texts [21]. Further analyses will also be conducted using propensity score based methods [22]. This will allow for quantification of bias and sensitivity analyses to explore robustness of the estimates to un-measured confounding. Secondary analyses based on utility weighted mRS (UW – mRS) will also be performed to provide estimates of effect size for future trials [23]. We propose to conduct multivariable modified Poisson regression and survival analyses for assessing relative risk of 30-day readmission, and hazard ratio for 30-day mortality respectively. Differences in the median scores for quality of life will be analyzed using quantile regression methods. Day-90 cost effectiveness analysis will be performed from payer’s perspective using Medicare weight reimbursement rates. Long-term (1 Year) costs and outcomes will be modeled using Markov modeling. Sensitivity analyses will be conducted to determine the robustness of the incremental cost effectiveness ratio. Finally, receiver operating curve based predictive scores will be developed for ICH patients who are likely to benefit from care at CSCs. This will help identify a subset of ICH patients optimally benefiting from HLOC.

### Sample size and power

The power and sample size calculations of the study have been conducted to satisfy the primary endpoint of 90-day mRS. We determined the 90-day distribution of mRS for ICH patients using published literature [24]. In addition, based on our prior work we estimate that approximately 60% ICH patients are managed at CSCs [9]. Therefore using a ratio of 1.5 for CSC vs. Non-CSC patients, a total of 1110 patients are needed to achieve 80% power to detect a change in log odds ratio of 0.3 with the significance level of 0.05 for a two-sided test [25]. Factoring in an attrition rate of 20% we plan to enroll 1375 patients (825 CSC patients and 550 non-CSC patients) over the period of 2.5 years, such that all follow up is completed within 3 years of study duration.

### Progress

**Fig. 2:**
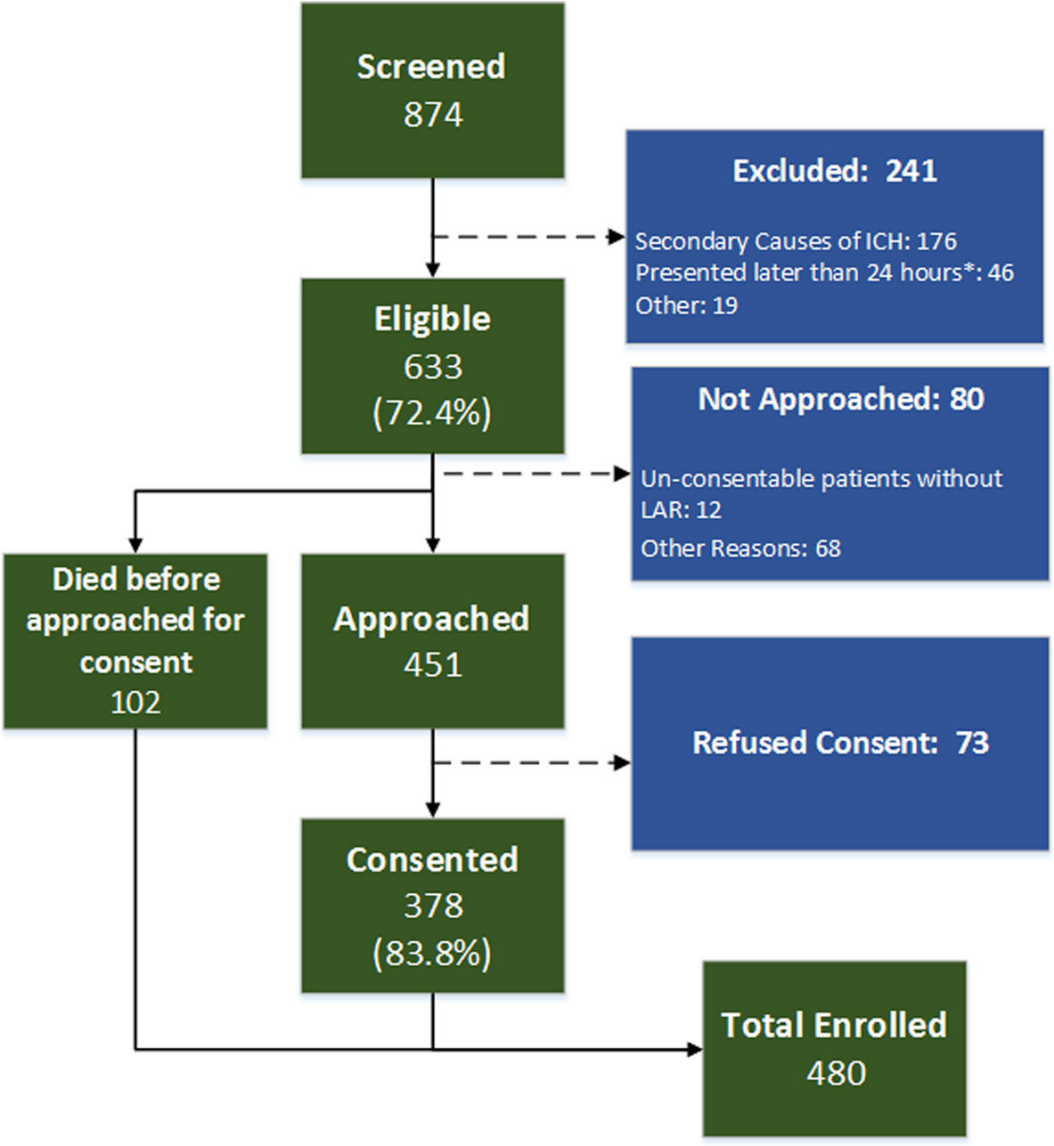
Flow diagram for screening, eligibility, approach, and consent for current status of EnRICH (combined for all participating sites)

Since initiation in September 2016, EnRICH has been operationalized at a total of nine hospitals in larger metropolitan areas of Houston, Austin, San Antonio, and Dallas.. Study start-up procedures are currently ongoing at two other LSS hubs. To-date, over 870 patients have been screened and 480 patients have been enrolled. The current loss to follow up rate for both the 30 and 90-day time points is less than 5%. Fig. 2 shows a flow diagram of the number and proportion of screened, approached, consented, and enrolled patients. Reasons for non-enrollment are also indicated. Since the approval and implementation of a verbal consent process in late September 2016, the refusal rate for study participation has declined from 41.0% to 16.2%.

## Discussion

EnRICH is a statewide cohort of ICH patients that will answer important questions regarding regionalization of care. The rationale of EnRICH stems from the gravity of the disease, lack of definitive treatment modalities for ICH patients, and current practice patterns in the absence of class I evidence. Though it remains critically important to explore novel management strategies for ICH treatment, it is perhaps even more crucial to ensure that the resources within current stroke care delivery systems are optimized to provide maximal benefit for ICH patients. On one hand, the implementation of evidence-based management practices are important, but at the same time, non-evidence based practices are necessary to be evaluated and eventually de-implemented. Parameters for management of ICH patients at a given level of care lack clarity and specificity, resulting in undue strain on systems of care, with probably little to no impact on patient-centered outcomes. Synthesized evidence supporting national guidelines and certifications should provide generalizable content and need to be contextually relevant. EnRICH will provide much needed data relevant to the changing and future paradigm of stroke care delivery in the US, in which an increasing number of stroke care facilities strive to achieve a certification status [26].

It is likely that decisions to transfer ICH patients for HLOC is an interplay of multiple patient, clinical, caregiver, physician, policy, and resource factors; with significant facilities-wide and regional variation. Recognizing the array of stakeholders in the transfer process, we conducted multiple brainstorming and focused group discussions with clinical trial design experts, analysts, stroke neurologists, neuro-intensivists, emergency care physicians, hospital administrators, members of committee for protection of human subjects, and patients and their care givers. All agreed that a trial in which patients were randomized to either stay at a certain hospital, or be transferred to a different one, was not feasible. Furthermore, there is a dearth of evidence that would establish criteria for transfer of brain hemorrhage patients to a higher level of care. The current guidelines provide extremely broad and non-precise parameters. We do hypothesize that transfer is beneficial for a certain subset of ICH patients, and believe that our work will help establish these parameters more precisely. Given this information, we continue to assess the feasibility of a step-wedge randomization design for the next phase of the study. The data from EnRICH will help us to design such a randomized trial. Therefore, despite the limitation of observational data, EnRICH is best suited to provide evidence leading to a better understanding of the impact of the level of care on outcomes for ICH patients. The design is also conducive to implementation at multiple sites, particularly those with limited resources for stroke research.

The results of the EnRICH study will have important implications for public policy and care provision, and may support evidence-based adaptation by healthcare systems. The current practice of indiscriminate patient transfer is not sustainable and is unlikely to translate into optimal societal benefit, particularly in non-urban populations with limited access to care. Economic analyses of EnRICH data will further inform rational decisions regarding the allocation of limited resources, and analyses of ICH patient outcomes - including patient quality of life - will provide direction to agencies responsible for hospital accreditation as they formulate standards and guidelines for care of ICH patients.

## Abbreviations

CI: Confidence interval
CSC: Comprehensive stroke center
EQ-5D: EuroQol – 5 Dimensions
GCS: Glasgow coma scale
HLOC: Higher level of care
ICH: Intracerebral Hemorrhage
IRB: Institutional review board
LSS: Lone star stroke
MoCA: Montreal cognitive assessment
mRS: Modified rankin scale
NICU: Neurological intensive care unit
NIHSS: National institutes of health stroke scale
PSC: Primary stroke center
QALY: Quality-adjusted life years
QOL: Quality of life
US: United States
UW-mRS: Utility weighted modified rankin scale
